# Chimeric Antigen Receptor–Immune Cell-Based Therapies for Clear Cell Renal Cell Carcinoma: Latest Advancements and Directions

**DOI:** 10.3390/cancers18132051

**Published:** 2026-06-24

**Authors:** Xuyuan Zhu, Yu Zhang, Yuxiang Chen, Shanda Li, Kun Wang, Tao Li, Xiaojie Ma, Zhuona Ni, Hongtao Jiang

**Affiliations:** 1 Department of Kidney Transplantation, The Second Affiliated Hospital of Hainan Medical University, Haikou 570311, China; 15988000501@muhn.edu.cn (X.Z.); 2303302046@muhn.edu.cn (Y.Z.); chenyuxiang1@shhmu.net (Y.C.); 2403302322@muhn.edu.cn (S.L.); hywk2025@muhn.edu.cn (K.W.); litao@shhmu.net (T.L.); 2 Department of Rehabilitation, The Second Affiliated Hospital of Hainan Medical University, Haikou 570311, China; maxiaojie@shhmu.net; 3 Department of Physiology, Zhejiang Chinese Medical University, Hangzhou 310053, China; 202411011411016@zcmu.edu.cn

**Keywords:** clear cell renal cell carcinoma, chimeric antigen receptor T cells, tumour microenvironment, immunotherapy, CAR-NK cells, CAR-NKT cells

## Abstract

Clear cell renal cell carcinoma (ccRCC) accounts for approximately 75% of renal cell carcinomas and is defined by near-universal VHL inactivation, leading to constitutive HIF stabilisation, metabolic reprogramming, and an immunologically distinct tumour microenvironment (TME). Although ccRCC is characterised by abundant immune infiltration, this paradoxically correlates with poor prognosis, reflecting a TME that imposes interconnected physical, immunological, and metabolic barriers to effective immunotherapy. Chimeric antigen receptor (CAR)-based therapies have revolutionised the treatment of haematological malignancies, but their translation to ccRCC has encountered substantial hurdles. The first-in-human trial targeting carbonic anhydrase IX (CAIX) was limited by on-target off-tumour toxicity and CAR immunogenicity—lessons that fundamentally reshaped the field. CD70 has since emerged as the dominant clinical target, expressed in over 80% of ccRCCs with a highly restricted normal tissue distribution. The phase I COBALT-RCC trial of CTX130, an allogeneic CRISPR-Cas9-edited CD70-directed CAR-T cell product, provided formal proof of concept, achieving disease control in 81.3% of heavily pretreated patients and a durable complete response now exceeding three years—the first such sustained remission reported for any CAR-T cell product in a solid malignancy. Nevertheless, the low frequency of durable responses and universal loss of CAR-T cell persistence by day 28 underscore that major barriers remain. Beyond CD70, the field has diversified across multiple platforms, including CAR–natural killer (NK) cells, CAR–natural killer T (NKT) cells, and CAR–macrophages, each offering distinct biological advantages. This review synthesises current knowledge of the ccRCC TME, the preclinical landscape of CAR-based therapies, and emerging clinical evidence from more than 30 registered trials. We discuss target antigens; engineering strategies to overcome TME barriers, including cytokine armouring, chemokine receptor co-expression, switch receptors, and metabolic reprogramming; and rational combination approaches. We argue that the convergence of optimised target selection, cellular engineering, combination strategies, and biomarker-driven trial design may ultimately improve outcomes for patients with ccRCC. However, achieving a cure remains an aspirational goal, and significant barriers must first be overcome.

## Simple Summary

Clear cell renal cell carcinoma (ccRCC) is the most common type of kidney cancer. It grows within a specialised tumour microenvironment (TME) that makes conventional immunotherapies less effective. CAR-T cell therapy has changed the treatment of blood cancers, but similar success has not yet been achieved in solid tumours such as ccRCC. This review summarises the latest developments in chimeric antigen receptor (CAR)-based immunotherapies for kidney cancer. It examines over thirty registered clinical trials and describes how different approaches—such as optimising the CAR design, engineering various immune cell types (including T cells and NK cells), and combining therapies—may help address the physical, immunological, and metabolic barriers created by the TME. The goal is to provide researchers and clinicians with an accessible overview of how these cellular therapies might move from laboratory research to clinical use.

## 1. Introduction

Clear cell renal cell carcinoma (ccRCC) accounts for approximately 75% of all renal cell carcinomas and represents one of the most immunologically distinct solid malignancies [1]. Unlike many other cancers, ccRCC is characterised by abundant immune infiltration, yet, paradoxically, this feature correlates with poor prognosis rather than improved outcomes [2]. This immunological paradox, combined with the tumour’s responsiveness to immune checkpoint blockade, has positioned ccRCC as a paradigm for understanding tumour–immune interactions [3]. However, despite advances in first-line combination regimens, most patients eventually progress, and durable complete responses remain rare [4,5,6]. The tumour microenvironment (TME) of ccRCC imposes multifaceted barriers that limit conventional immunotherapies: physical barriers erected by cancer-associated fibroblasts and abnormal vasculature, immunological barriers driven by T cell exhaustion and myeloid suppression, and metabolic barriers arising from hypoxia, acidosis, and nutrient competition [7]. These interconnected obstacles demand therapeutic strategies that go beyond checkpoint blockade alone.

Chimeric antigen receptor (CAR)-based therapies have revolutionised the treatment of haematological malignancies, but their translation to solid tumours, including ccRCC, has encountered substantial hurdles [8]. The first-in-human trial targeting carbonic anhydrase IX (CAIX) demonstrated feasibility but was limited by on-target off-tumour toxicity and CAR immunogenicity [9,10]. These lessons reshaped the field, shifting the focus toward more restrictive targets, affinity-tuned designs, and strategies to reduce immunogenicity.

CD70 has emerged as an attractive ccRCC target, overexpressed in over 80% of cases with a restricted normal tissue distribution [11]. The phase I COBALT-RCC trial of CTX130, an allogeneic CRISPR-Cas9-edited CD70-directed CAR-T product, provided proof of concept for CAR therapy in a solid tumour [12]. Nevertheless, durable responses remain rare and CAR-T persistence is limited [13,14,15].

Beyond CD70, the field has diversified across platforms: CAR-NK cells offer off-the-shelf availability [16]; CAR-NKT cells combine CAR-dependent and innate recognition [17]; and CAR–macrophages provide phagocytosis-centred TME remodelling [18].

This review synthesises current knowledge of the ccRCC TME, preclinical CAR-based platforms, and emerging clinical evidence. We discuss major targets (B7-H3, CAIX, CD70), engineering strategies, registered trials, and future directions.

## 2. The Biological Basis of Clear Cell Renal Cell Carcinoma

### 2.1. Tumour Cells

ccRCC is defined by the near-universal inactivation of the von Hippel–Lindau (VHL) tumour suppressor gene, occurring in over 90% of sporadic cases. This genetic lesion, together with chromosome 3p loss, represents the initiating event in ccRCC ontogeny and fundamentally shapes the TME [19]. The TRACERx Renal studies demonstrated that 3p loss occurs early through chromothripsis, simultaneously disrupting VHL, PBRM1, SETD2, and BAP1 [20].

VHL loss leads to the constitutive stabilisation of hypoxia-inducible factors HIF-1α and HIF-2α, which orchestrate a broad transcriptional programme encompassing angiogenesis, metabolic reprogramming, and immune modulation [21]. HIF-1α predominantly regulates glycolytic enzymes and pro-survival pathways, whereas HIF-2α drives stemness and immunoregulatory gene expression [22]. Downstream targets include vascular endothelial growth factor (VEGF), CAIX, CD70, and programmed death ligand 1 (PD-L1), each contributing to the distinctive architecture of the ccRCC TME [23,24]. Beyond HIF-dependent effects, VHL exerts non-canonical functions that explain why VHL loss alone is insufficient for tumourigenesis [25].

Metabolic reprogramming is the second pillar of ccRCC. Tumour cells exhibit the Warburg effect (aerobic glycolysis) and glutamine addiction, while lactate secretion acidifies the microenvironment to pH < 6.8, suppressing T cell function and promoting Treg expansion [26,27]. This metabolic rewiring not only sustains tumour cell proliferation but actively sculpts an immunosuppressive niche. The identification of metabolic vulnerabilities, including a dependence on the mevalonate pathway and fatty acid synthesis, has opened up new therapeutic avenues [28].

The antigenic landscape of ccRCC reflects the HIF-driven expression of CD70 (>80% of cases) and CAIX, both attractive CAR targets [29]. However, intratumoural heterogeneity, revealed by the TRACERx Renal studies, demonstrates that distinct subclones within the same tumour exhibit divergent antigen profiles, creating spatially variable immune recognition landscapes that complicate therapeutic targeting and facilitate antigen escape under selective pressure [30]. Single-cell analyses have further revealed that tumour cells from different regions display distinct transcriptional programmes, with implications for both immune evasion and therapy resistance [20]. Regarding the impacts of VHL-HIF signalling on CAR cells, HIF activation directly affects CAR cell trafficking, persistence, and function: (1) aberrant CXCR4/SDF-1α gradients may misdirect trafficking [31]; (2) HIF-driven CD39/CD73 expression generates adenosine that suppresses CAR cell proliferation [32]; (3) HIF-2α upregulates PD-L1, promoting exhaustion [33]; and (4) hypoxia and acidosis impair CAR cell metabolic fitness. These links suggest that HIF-2α inhibitors (e.g., belzutifan) could be synergised with CAR therapy [33].

### 2.2. Immune Cells

The ccRCC immune microenvironment is characterised by abundant immune infiltration, yet, paradoxically, high T cell abundance correlates with poor prognosis [34]. Single-cell technologies resolved this paradox by revealing functional continua. CD8+ T cells progress from progenitor (TCF7+, TCF1+) to terminally exhausted (TOX+, PD-1+, TIM-3+, LAG-3+) states, with terminal exhaustion being epigenetically locked and resistant to reinvigoration [35,36]. This exhaustion continuum is regulated by transcription factors including TOX, EOMES, and BATF, alongside chromatin accessibility changes and DNA methylation at effector loci. Notably, terminally exhausted CD8+ T cells are highly clonal, indicating repeated antigen exposure, and represent a substantial proportion of the T cell repertoire in advanced disease [37].

Tumour-associated macrophages (TAMs) extend beyond M1/M2. SPP1+ TAMs correlate with poor prognosis and ICB resistance, whereas C1Q+ and TREM2+ populations regulate immunity and are associated with recurrence [38]. C1Q+ TAMs participate in immune regulation and apoptotic cell clearance, whereas TREM2+ populations are associated with disease recurrence. IL-1β-expressing macrophages reside at tumour-normal interfaces, co-localising with epithelial–mesenchymal transition (EMT)-high tumour cells to facilitate invasion. M2-polarised TAMs predominate in advanced disease and interact with CD8+ T cells through multiple immune checkpoint ligands, including PD-L1, CD80, CD86, and CD155, contributing to T cell exhaustion [34]. The dynamic shift from pro-inflammatory to anti-inflammatory TAM phenotypes with disease progression underscores the evolving nature of the immune microenvironment [39].

Cancer-associated fibroblasts (CAFs), the predominant stromal population, exhibit functional heterogeneity rooted in diverse cellular origins [40]. Myofibroblastic CAFs (myCAFs) produce collagen and fibronectin, driving matrix stiffness and physical barrier formation [41]. Inflammatory CAFs (iCAFs) secrete IL-6, CXCL12, and LIF, establishing a chemokine barrier that excludes CD8+ T cells from tumour nests while recruiting immunosuppressive populations. Antigen-presenting CAFs (apCAFs) express MHC class II molecules yet lack co-stimulatory ligands, inducing T cell anergy [42]. Single-cell analyses of recurrent ccRCC have revealed expanded CAF populations with upregulated GAL-1, which promotes CD8+ T cell apoptosis and contributes to diminished immunotherapy efficacy. The spatial organisation of CAFs relative to tumour cells and immune infiltrates critically influences clinical outcomes [42].

B cells within tertiary lymphoid structures (TLSs) contribute to local immunity. Mature intratumoural TLSs correlate with improved ICB responses, whereas peritumoural immature TLSs are associated with poor outcomes [43,44]. NK cells and dendritic cells (DCs) complete the immune landscape, with tumour-infiltrating NK cells exhibiting impaired cytotoxicity despite preserved cytokine production and DC subsets demonstrating context-dependent functional plasticity [45].

### 2.3. Extracellular Matrix

The extracellular matrix (ECM) in ccRCC is not a passive scaffold but an active participant in tumour progression. Remodelling driven by CAFs and matrix metalloproteinases (MMPs) alters collagen organisation, increases tissue stiffness, and creates physical barriers that impede immune cell infiltration [46].

Abnormal vasculature represents a specialised ECM compartment with profound functional consequences. VHL-HIF-VEGF signalling produces structurally aberrant vessels characterised by tortuosity, hyperpermeability, and poor pericyte coverage [47]. These vessels impair blood flow, creating regions of hypoxia and acidosis, while downregulating adhesion molecules such as ICAM-1 and VCAM-1, thereby impeding lymphocyte extravasation [48]. Endothelial cells in ccRCC additionally express indoleamine 2,3-dioxygenase (IDO), depleting tryptophan and generating immunosuppressive kynurenine. The resulting vascular dysfunction establishes a physical and biochemical barrier that limits both drug delivery and immune cell trafficking. Anti-angiogenic therapy can transiently normalise this abnormal vasculature, creating a therapeutic window that enhances immune cell infiltration [49,50].

Exosomes have emerged as critical mediators of intercellular communication within the TME. These 30–150 nm extracellular vesicles carry diverse cargo including proteins, mRNAs, miRNAs, and lncRNAs, transferring information from donor to recipient cells [51]. Tumour-derived exosomes promote angiogenesis through VEGF and miR-27a delivery, induce M2 macrophage polarisation, and directly suppress NK cell function via TGF-β and immunomodulatory miRNAs [52]. Exosomal lncARSR mediates sunitinib resistance by transferring resistance phenotypes between tumour cells, establishing a positive feedback loop that amplifies drug resistance. CAF-derived exosomal miR-590-3p confers radioresistance through the activation of PI3K/AKT signalling. The stability of exosomal nucleic acids in body fluids positions them as attractive liquid biopsy biomarkers for the monitoring of treatment responses and resistance evolution [53].

### 2.4. Therapeutic Strategies

Current first-line standards for advanced ccRCC combine PD-1/PD-L1 inhibitors with VEGFR-TKIs or CTLA-4 blockade. HIF-2α inhibitors (belzutifan) have entered clinical use for VHL-associated ccRCC [54,55].

The TME imposes multifaceted resistance mechanisms that limit therapeutic durability. Primary resistance manifests as immune-excluded or immune-desert phenotypes, often driven by CAF-rich stroma, myeloid cell dominance, or antigen presentation defects [56]. PBRM1-mutated tumours, which account for 30–40% of ccRCCs, exhibit reduced immune infiltration and diminished responses to ICB, whereas BAP1-mutated tumours show increased T cell inflammation [57]. Acquired resistance involves T cell exhaustion, alternative checkpoint upregulation (TIM-3, LAG-3, TIGIT), and suppressive population expansion [58].

Emerging strategies aim to overcome these barriers through rational combination. Vascular normalisation windows following anti-angiogenic therapy create opportunities to enhance immune cell infiltration when timed with ICB [59]. CAF-targeting approaches, including FAP-directed CAR-T cells and TGF-β inhibitors, seek to dismantle physical and chemokine barriers [60]. TAM repolarisation using CSF-1R inhibitors or TLR agonists aims to shift the myeloid balance from immunosuppressive to pro-inflammatory states. TLS-inducing strategies, leveraging cytokines such as LIGHT or IL-33, hold promise for generating local immune hubs that support sustained antitumour responses. Metabolic interventions targeting lactate transport or glutamine metabolism may alleviate nutrient competition and restore T cell function [60].

**Figure 1:**
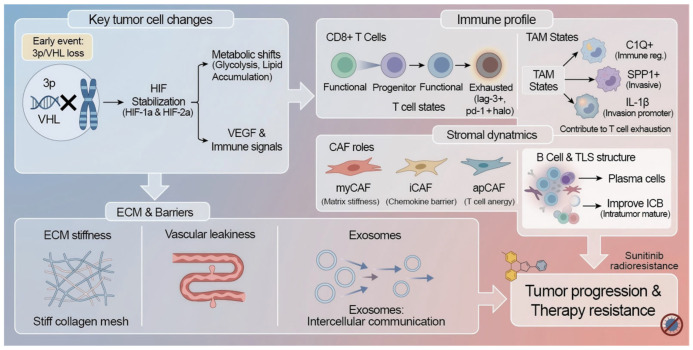
Overview of the RCC tumour microenvironment and mechanisms of therapy resistance. This figure illustrates the three interconnected barriers (physical, immunological, and metabolic) imposed by the ccRCC TME and highlights the key cellular players—including CAFs, TAMs, and exhausted T cells—that drive therapy resistance. Abbreviations: CAF, cancer-associated fibroblast; ECM, extracellular matrix; HIF, hypoxia-inducible factor; ICB, immune checkpoint blockade; LAG-3, lymphocyte activation gene 3; PD-1, programmed cell death protein 1; RCC, renal cell carcinoma; TAM, tumour-associated macrophage; TIGIT, T cell immunoreceptor with Ig and ITIM domains; TLS, tertiary lymphoid structure; VHL, von Hippel–Lindau.

A systems-level model of CAR cell failure in ccRCC arises from three synergistic barriers [61]. Physical barriers (CAF-rich stroma, abnormal vasculature) limit CAR cell infiltration. Immunological barriers (exhaustion, Tregs, MDSCs) suppress effector function. Metabolic barriers (hypoxia, acidosis, nutrient competition) impair metabolism and survival. These barriers form a self-reinforcing loop: poor infiltration reduces activation, enabling tumour progression and further TME remodelling. Hence, future directions centre on precision stratification by TME architecture—using four integrated proteogenomic subtypes (CD8+ inflamed, CD8− inflamed, VEGF-high immune desert, metabolic immune desert) and multiparametric biomarkers (PD-L1, TLS, PBRM1/BAP1/SETD2, T cell clonality) to guide combination therapies [62,63]. As single-cell and spatial technologies refine TME understanding, ecosystem-level modulation tailored to individual tumour architectures is approaching clinical reality [64]. Effective ccRCC treatment must therefore disrupt multiple barriers simultaneously, not single mechanisms in isolation.The interconnections between these three barriers and the key cellular players driving them are integrated schematically in Figure 1, which provides a conceptual framework for understanding the multifaceted TME landscape in ccRCC.

## 3. CAR-Based Therapies in RCC

### 3.1. CAR-T Cells

CAR-T cell therapy has revolutionised the treatment of haematologic malignancies, but its translation to solid tumours, including RCC, has encountered substantial hurdles [65]. These include the identification of suitable target antigens, on-target off-tumour (OTOT) toxicity, an immunosuppressive tumour TME, and poor T cell persistence. Recent preclinical advances have begun to address these challenges through novel target discovery, affinity engineering, combination strategies, and intrinsic T cell engineering. This section synthesises these developments, focusing on the three most extensively investigated targets, B7-H3, CAIX, and CD70, alongside emerging antigens and innovative engineering approaches [66].

B7-H3, a type I transmembrane protein belonging to the B7 immune checkpoint family, has emerged as an attractive CAR-T target due to its aberrant overexpression on tumour cells and minimal expression in normal tissues [67]. In RCC, bioinformatics analysis combined with immunohistochemical validation has demonstrated that B7-H3 is highly expressed in both tumour tissues and RCC cell lines, whereas its expression is undetectable in normal renal parenchyma. Elevated B7-H3 levels correlate with poor prognosis, suggesting functional relevance in disease progression [68]. To target this antigen, second-generation CAR-T cells incorporating either CD28 or 4-1BB co-stimulatory domains have been developed [69]. Following lentiviral transduction, CAR expression was confirmed by flow cytometry, with reported transduction efficiencies of approximately 40% [70]. In co-culture experiments, B7-H3-directed CAR-T cells exhibited potent and specific cytotoxicity against RCC cell lines, with killing activity correlating positively with the effector-to-target ratio. Functional characterisation revealed robust cytokine production, including IFN-γ, IL-2, and granzyme B, alongside elevated lactate dehydrogenase (LDH) release [70]. Importantly, both CD28- and 4-1BB-based CAR constructs demonstrated comparable antitumour activity in vitro. The therapeutic potential was further validated in vivo using metastatic and orthotopic RCC xenograft models, where the systemic administration of B7-H3 CAR-T cells significantly inhibited tumour growth compared to untransduced T cell controls. These findings establish B7-H3 as a valid immunotherapeutic target in RCC [69,70]. To date, B7-H3 CAR-T cells have been evaluated only in in vitro and in vivo preclinical models and have not yet entered clinical trials for RCC (see Section 4 for registered clinical trials of other targets).

CAIX is overexpressed in over 90% of ccRCC cases and represents one of the most extensively studied CAR-T targets. However, the first-in-human clinical trial using the high-affinity G250 CAR-T cells was limited by OTOT toxicity, specifically dose-limiting cholangitis due to low-level CAIX expression on biliary epithelial cells. This safety hurdle rendered CAIX undruggable for nearly a decade [71]. Recent affinity engineering has revisited this target. Using direct stochastic optical reconstruction microscopy (dSTORM) at a single-molecule resolution, researchers identified high CAIX density in ccRCC patient samples but low-density expression in healthy bile duct tissues [72]. A Tet-On doxycycline-inducible CAIX-expressing cell line was established to systematically interrogate the relationship between antigen density and CAR-T activation. Through affinity fine-tuning, the low-affinity/high-avidity G9 CAR-T demonstrated a substantially wider therapeutic window compared to the high-affinity G250 variant. In patient-derived organotypic tumour spheroid (PDOTS) ex vivo cultures, G9 CAR-T cells exhibited superior efficacy, enhanced migration, and robust cytokine release. In orthotopic RCC mouse models, G9 CAR-T cells achieved better tumour control than G250, successfully mitigating OTOT toxicity and rendering CAIX a druggable target once again [72,73].

Recognising that CAR-T monotherapy faces substantial barriers from the immunosuppressive TME, several groups have explored rational combination strategies pairing CAIX-directed CAR-T cells with tyrosine kinase inhibitors (TKIs) [74]. The combination with sunitinib demonstrated synergistic antitumour effects in both subcutaneous xenograft and lung metastasis models of human RCC. Mechanistically, sunitinib exerted dual benefits: it upregulated CAIX expression on tumour cells, potentially enhancing CAR-T recognition, and reduced the frequency of myeloid-derived suppressor cells (MDSCs) in the TME [75]. These changes led to enhanced CAR-T cell proliferation, infiltration, and persistence, translating into a significantly reduced tumour burden and prolonged survival [76]. Similarly, cabozantinib enhanced CAIX-specific CAR-T efficacy in orthotopic and subcutaneous RCC models. Beyond promoting CAR-T infiltration and reducing exhaustion markers, cabozantinib remodelled the TME by decreasing the TAM abundance and inhibiting polarisation toward the immunosuppressive M2 phenotype. The combination also disrupted the PD-1/PD-L1 axis, evidenced by reduced PD-L1 expression on tumour cells and decreased PD-1 on CAR-T cells [77]. An orthogonal strategy involves engineering CAR-T cells to actively remodel the TME. The immune-restoring (IR) CAR G36-PDL1 construct, a CAIX-directed CAR-T cell engineered to secrete anti-PD-L1 monoclonal antibody, was tested in a humanised ccRCC orthotopic mouse model reconstituted with human leukocytes. Compared to conventional CAR-T cells, G36-PDL1 CAR-T cells exerted potent antitumour effects, restored active immunity by promoting cytotoxic killing, reduced immunosuppressive populations (M2 macrophages and exhausted CD8+ T cells), and enhanced T follicular helper (Tfh)–B cell crosstalk [78]. The choice of co-stimulatory domain significantly influences CAR-T function, particularly when combined with immune checkpoint blockade (ICB). A systematic comparison of CD28-based versus 4-1BB-based anti-CAIX CAR-T cells revealed that CD28-based CARs releasing anti-PD-L1 antibodies exhibited superior antitumour activity in orthotopic models, achieving greater reductions in tumour volume and weight while preventing metastasis. Importantly, no hepatotoxicity or nephrotoxicity was observed at therapeutic doses, providing a safety foundation for clinical translation [78,79]. A potential cautionary note arises from the regulatory interaction between CAIX and cyclooxygenase-2 (COX-2). COX-2 blockade with celecoxib led to the significant downregulation of CAIX expression, and the combination of celecoxib with anti-CAIX CAR-T therapy impaired the CAR-T cytotoxicity potential in a CAIX density-dependent manner. These findings suggest that COX-2 inhibitors should be avoided in combination regimens with CAIX-targeted therapies [80]. Following affinity re-engineering, CAIX-directed CAR-T cells have re-entered early-phase clinical evaluation (Section 4).

CD70, a member of the tumour necrosis factor family, has emerged as one of the most promising immunotherapeutic targets in RCC [81]. It was found to be expressed in 80.9% of cases, with high expression associated with an advanced T stage and reduced overall survival. Single-cell transcriptomics revealed that CD70 is predominantly expressed on immune cells, particularly T cells, within the TME, highlighting the complexity in targeting this antigen [82]. Nevertheless, restricted expression in normal tissue positions CD70 as an attractive CAR-T target. Multiple anti-CD70 CAR constructs have been developed using distinct antigen-binding domains. Nanobody-based CAR-T cells demonstrated superior functionality compared to scFv-based constructs, producing significantly higher levels of IL-2, IFN-γ, and TNF-α upon co-culture. In xenograft models, nanobody-based CAR-T cells led to the eradication of RCC tumours, with significantly higher human T cell expansion after infusion. Mice treated with VHH CAR-T remained tumour-free upon re-challenge, demonstrating durable antitumour immunity [83]. A subset of ccRCC metastases may show heterogeneous or reduced CD70 expression, potentially enabling antigen escape and acquired resistance [84]. Strategies to mitigate this include combinatorial targeting (e.g., CD70 plus CAIX or B7-H3) and CAR-NKT platforms that retain natural killer receptor-mediated killing independently of CD70 expression (discussed in Section 3.2 and Section 3.3). Unlike in haematologic malignancies, where antigen loss is a documented escape mechanism, no longitudinal clinical data exist demonstrating CD70, CAIX, or B7-H3 downregulation after CAR cell pressure in RCC. This remains an important knowledge gap for future study. A unique challenge in targeting CD70 is its expression on activated T cells, creating the potential for CAR-T cell fratricide. Through systematic characterisation, researchers identified CAR constructs that enabled production despite CD70 expression, as CAR expression masked CD70 detection in cis and provided protection from fratricide. Two distinct classes of CAR-T cells were identified, differing in memory phenotype, activation status, and cytotoxic activity. Highly functional allogeneic CD70 CAR-T cells were successfully produced at a large scale through TALEN-based gene editing to eliminate the T cell receptor, paving the way for off-the-shelf products [85]. To enhance the CAR-T efficacy, PARP inhibition with olaparib was combined with CD70 CAR-T cells in RCC xenograft models. The combination led to superior tumour regression, improved survival, and increased CAR-T cell persistence. Mechanistically, olaparib induced DNA damage and cytosolic double-stranded DNA accumulation, activating the cGAS-STING pathway and increasing the expression of the chemokines CCL5 and CXCL10, which promoted CD8+ CAR-T cell infiltration into the TME [86]. An innovative approach to overcoming TME barriers involves the use of gamma delta (γδ) T cells. ADI-270, an allogeneic γδ CAR-T cell product targeting CD70+ cancers, engineered with a third-generation CAR based on the natural CD27 receptor, incorporates a dominant negative TGFβ receptor II (dnTGFβRII) for functional armouring. Patient-derived RCC organoids have provided functional validation for CD70 CAR-T cells. A modified three-dimensional culture system established 33 RCC organoid lines preserving parental tumour characteristics. Co-culture of CD70 CAR-T cells with CD70-positive RCC organoids resulted in significantly increased IFN-γ and TNF-α production, enhanced CAR-T proliferation, and elevated cleaved caspase-3 levels, with no effects on CD70-negative normal kidney organoids [87]. CD70 is the most clinically advanced target for CAR-based therapy in RCC, with multiple phase I/II trials ongoing (see Section 4 for detailed clinical outcomes).

Beyond the well-characterised targets, several additional antigens have shown promise. DNAJB8, a cancer/testis antigen driving cancer stem-like cells, was targeted with a second-generation CAR recognising the HLA-A*24:02/DNAJB8 peptide complex. B10 CAR-T cells demonstrated specific cytotoxicity against RCC and osteosarcoma cells in vitro and in vivo [88]. ROR2, a receptor tyrosine kinase-like orphan receptor, was evaluated using affinity-matured second-generation CAR-T cells. ROR2-CAR-T cells induced rapid tumour remission in ccRCC xenografts, extending the median overall survival from 52 to 84 days (*p* = 0.0035) [89]. Safety assessments revealed no OTOT toxicity, and ROR2 expression in normal adult tissues was highly restricted. c-Met, overexpressed in 97% of papillary RCC (PRCC) clinical specimens, was targeted with third-generation CAR-T cells. In an orthotopic PRCC model, a single dose induced complete regression in approximately 60% of mice. Combination with axitinib synergistically enhanced the low-dose CAR-T efficacy, mirroring the TKI combination strategies described for CAIX [90].

Beyond target selection, the direct engineering of CAR-T cells to overcome metabolic and microenvironmental barriers represents an emerging frontier. Enforced expression of the glucose transporter GLUT1 significantly improved tumour control in RCC, leukaemia, and glioblastoma models. GLUT1 promoted a T stem cell-like memory phenotype, upregulated glycolytic and oxidative phosphorylation gene programmes, and reduced exhaustion markers. Notably, overexpression of the higher-affinity transporter GLUT3 did not confer similar benefits, indicating a specific effect dependent on GLUT1’s unique properties [91]. An orthogonal approach involves engineering CAR-T cells to secrete an NGF-neutralising scFv, achieving tumour immunosympathectomy. This strategy enhanced tumour suppression by inhibiting terminal exhaustion in tumour-infiltrating CD8+ T cells and preventing M2 macrophage polarisation [92]. These engineering strategies—metabolic optimisation and microenvironment remodelling—complement target-specific approaches by addressing the fundamental barriers of nutrient competition and sympathetic nerve-mediated immunosuppression.

Traditional two-dimensional culture systems fail to recapitulate the complex architecture of the RCC TME, while animal models are costly and lack human immune components. A rapid and economical three-dimensional microphysiological “RCC-on-a-chip” system was developed using RCC spheroids embedded in a collagen ECM. Notably, 3D culture induced a gene expression profile more closely resembling that of primary human RCC tumours compared to 2D culture. The platform successfully modelled antigen-specific immunotherapy: ROR1-directed CAR-T cells infiltrated the collagen ECM and mediated the targeted killing of ROR1+ RCC spheroids. This platform provides a physiologically relevant, quantitative, and cost-effective tool for preclinical CAR-T evaluation [93].

Preclinical studies have established a robust foundation for CAR-T cell therapy in RCC, with three major targets, B7-H3, CAIX, and CD70, advancing through systematic optimisation. CAIX exemplifies how affinity engineering can resurrect a previously failed target, while CD70 demonstrates rapid translation toward clinical evaluation, including allogeneic γδT products. Emerging targets such as ROR2 and c-Met address distinct RCC subtypes, and intrinsic T cell engineering strategies offer solutions to metabolic and microenvironmental barriers [94]. Collectively, these advances support the continued clinical development of CAR-T cell therapies for patients with RCC.

### 3.2. CAR-NK Cells

NK cells offer distinct advantages over T cell-based therapies for solid tumours, including “missing-self” recognition, intrinsic antibody-dependent cellular cytotoxicity (ADCC) via CD16, and a favourable safety profile with low risks of cytokine release syndrome or graft-versus-host disease [95,96]. Unlike CAR-T cells, CAR-NK cells can be derived from multiple sources, including allogeneic donors, cord blood, the NK-92 cell line, and induced pluripotent stem cells (iPSCs), enabling “off-the-shelf” manufacturing [16,97]. Furthermore, NK cells lack the potential to induce graft-versus-host disease, as they do not require T cell receptor signalling for activation [98]. These properties position CAR-NK cells as an attractive alternative or complement to CAR-T therapy for RCC. This section summarises preclinical advances in CAR-NK cell engineering for RCC, focusing on target selection, combination strategies, and emerging platforms such as CAR-NKT cells [99].

A third-generation CAR targeting epidermal growth factor receptor (EGFR) was developed and transduced into NK-92 cells via lentiviral infection. EGFR-specific CAR-NK-92 cells demonstrated the potent and specific lysis of RCC cell lines in an EGFR-dependent manner [100]. To overcome the immunosuppressive TME, the combination of cabozantinib, a multi-target tyrosine kinase inhibitor, with EGFR-CAR-NK-92 cells was evaluated. Cabozantinib treatment increased EGFR expression and decreased PD-L1 membrane surface expression on RCC cells, thereby enhancing the susceptibility of tumour cells to CAR-NK-mediated killing in vitro. Furthermore, the combination therapy exhibited synergistic therapeutic efficacy in human RCC xenograft mouse models, with significantly improved tumour control compared to either monotherapy [101,102]. These findings establish that the cabozantinib-mediated modulation of target antigen expression and immune checkpoint downregulation can potentiate CAR-NK function, providing a rational combination strategy for RCC.

Similar combination principles have been applied to other targets. A CAIX-specific third-generation CAR was transduced into NK-92 cells, generating CAIX-CAR-NK92 cells that specifically recognised CAIX-positive RCC cell lines (Ketr-3, OSRC-2) and released significant levels of IFN-γ, perforin, and granzyme B. To enhance the therapeutic efficacy, the proteasome inhibitor bortezomib was evaluated in combination with CAIX-CAR-NK92 cells. Bortezomib pretreatment enhanced the sensitivity of RCC cells to CAR-NK-mediated killing in vitro. In NOD/SCID mouse models bearing Ketr-3 human RCC xenografts, the combination of bortezomib and CAIX-CAR-NK92 cells significantly suppressed tumour growth compared to either monotherapy alone, with nearly complete tumour eradication observed in the combination group [103]. Beyond NK-92 cell lines, efforts have focused on engineering primary NK cells and related platforms. A clonal NK cell line expressing an HER2-specific CAR was generated following GMP-compliant procedures [104]. The CAR construct, based on the ErbB2-specific antibody FRP5 and harbouring CD28 and CD3ζ signalling domains (CAR 5.28.z), was stably expressed in NK-92 cells. NK-92/5.28.z cells efficiently lysed ErbB2-expressing tumour cells in vitro and exhibited serial target cell killing. In vivo, specific recognition and antitumour activity were retained, resulting in the selective enrichment of NK-92/5.28.z cells in orthotopic breast carcinoma xenografts and the reduction of pulmonary metastasis in an RCC model. Importantly, γ-irradiation, a potential safety measure for clinical application, prevented NK cell replication while preserving antitumour activity [105].

An innovative platform combining the properties of NK and T cells involves CAR-engineered natural killer T (CAR-NKT) cells [106]. A clinically guided protocol was developed for generating human CD70-directed allogeneic CAR-NKT cells (AlloCAR70-NKT) from cord blood CD34+ haematopoietic stem and progenitor cells (HSPCs). The protocol encompasses five sequential ex vivo differentiation stages: HSPC engineering, HSPC expansion, NKT differentiation, NKT deep differentiation, and NKT expansion. Using this platform, high-purity and high-yield AlloCAR70-NKT cells, theoretically up to 1 × 10^12^ cells, can be stably generated from a single cord blood unit, sufficient to treat thousands of patients. Functional evaluation demonstrated that AlloCAR70-NKT cells exert antitumour effects not only through CAR-dependent pathways but also via CAR-independent mechanisms mediated by natural killer receptors (NKRs) such as NKG2D and DNAM-1. Notably, these cells retained substantial cytotoxic activity even against CD70-low or CD70-knockout tumour cells, suggesting that CAR-NKT cells may overcome antigen escape, a major limitation of CAR-T therapy [107]. This protocol provides a clinically translatable, scalable, and standardised platform for developing “off-the-shelf” allogeneic cell immunotherapies for RCC and other CD70-positive malignancies.

Collectively, these preclinical studies establish CAR-NK and CAR-NKT cells as promising platforms for RCC immunotherapy. Key advances include the validation of multiple targets (EGFR, CAIX, ErbB2, CD70), rational combination strategies with TKIs (cabozantinib) and proteasome inhibitors (bortezomib), and the development of scalable manufacturing protocols for allogeneic products. The unique biology of NK cells, including CAR-independent killing via natural killer receptors and a favourable safety profile, positions CAR-NK platforms as attractive candidates for clinical translation, either as standalone therapies or in combination with existing treatment modalities for RCC.

### 3.3. Other CAR-Engineered Cells

γδ CAR-T cells are a distinct platform. Beyond conventional αβ CAR-T cells, γδ T cells offer unique advantages for allogeneic therapy, including innate-like recognition through their γδ T cell receptor (TCR) and a low risk of graft-versus-host disease (GVHD) [108]. ADI-270, an allogeneic γδ CAR-T cell product targeting CD70, engineered with a dominant-negative TGFβ receptor II, has entered phase I/II clinical evaluation for relapsed/refractory ccRCC (NCT06480565). A separate Vδ1 CAR-T cell study (NCT07113977) is also ongoing. These platforms represent a growing subset of CAR-T therapies that bridge innate and adaptive immunity.

Macrophages possess intrinsic tumour-homing capabilities, high phagocytic activity, and the ability to penetrate the immunosuppressive TME [109]. However, CAR-M therapy has been limited by phenotypic re-domestication—the tendency of adoptively transferred macrophages to switch from a proinflammatory (M1) to an immunosuppressive (M2) phenotype within the TME. To address this challenge, researchers developed an in situ-engineered chimeric interleukin-2 signalling receptor (CSR) that enables the controllable manipulation of the proinflammatory phenotype of CAR-Ms [110]. Customised lipid nanoparticles (LNPs) were developed to efficiently deliver dual circular RNAs (circRNAs) into macrophages, generating CSR-functionalised CAR-Ms. Upon stimulation with exogenous IL-2, the synthetic IL-2 receptor activates downstream TLR4 signalling pathways, inducing M1 polarisation and potent antitumour functions. A biomatrix hydrogel system enabled the localised and sustained co-delivery of LNPs and IL-2 into the subrenal capsule or postoperative tumour cavity. In orthotopic RCC mouse models, this combinatory strategy remodelled the immunosuppressive TME, promoted tumour regression, prevented postoperative recurrence, and suppressed lung metastasis. Mechanistically, CSR-engineered CAR-Ms enhanced antigen-specific phagocytosis, secreted proinflammatory cytokines, and reinvigorated CD8^+^ T cell activity. Therapeutic efficacy was further validated in a humanised patient-derived xenograft (PDX) model [111]. These findings establish that the proinflammatory phenotype of CAR-Ms can be modulated by a synthetic IL-2 receptor, offering a promising immunotherapeutic strategy for RCC with broad applicability to other solid malignancies.

iNKT cells represent another promising platform for CAR-based immunotherapy due to their non-MHC-restricted recognition and low risk of graft-versus-host disease (GVHD) [112]. However, their clinical application has been limited by low frequencies in peripheral blood and poor in vivo persistence. To overcome these barriers, researchers developed an optimised ex vivo expansion protocol using α-galactosylceramide (α-GC) combined with a cytokine cocktail (IL-2, IL-4, GM-CSF, and IL-21), generating high-purity, clinically scalable iNKT cells from healthy donor peripheral blood mononuclear cells within three weeks [113]. Notably, IL-21 significantly increased the frequency of CD62L^+^ central memory-like iNKT cells, a phenotype associated with enhanced long-term persistence and antitumour activity. These iNKT cells were further engineered to express a second-generation B7H3-targeting CAR with or without constitutive IL-21 expression. Mechanistically, IL-21 co-expression promoted STAT3 phosphorylation and conferred resistance to cytokine withdrawal-induced apoptosis. In NCG mouse models of subcutaneous, orthotopic, and metastatic renal cancer, B7H3-IL21.CAR-iNKT cells demonstrated significantly prolonged in vivo persistence, superior tumour suppression, and extended tumour-free survival compared to CAR-iNKT cells without IL-21, without observable cytokine-related adverse events or uncontrolled proliferation [114]. These findings establish that IL-21 armouring enhances the persistence and therapeutic efficacy of B7H3 CAR-iNKT cells, providing a promising off-the-shelf cellular immunotherapy strategy for solid tumours.

CD70-directed AlloCAR70-NKT cells have been developed as a next-generation platform addressing multiple limitations of conventional CAR-T therapy. Generated from haematopoietic stem and progenitor cells using a clinically guided, feeder-free culture method, these cells expand robustly with high purity (>99%) and naturally lack CD70 expression, eliminating the fratricide risk without requiring CD70 knockout [107]. AlloCAR70-NKT cells exhibit potent cytotoxicity against primary and metastatic RCC through both CAR- and natural killer receptor (NKR)-mediated mechanisms (including NKG2D and DNAM-1), enabling the effective targeting of CD70-low and CD70-negative tumours—a critical advantage for overcoming antigen escape [115]. Furthermore, they selectively deplete immunosuppressive CD1d^+^ tumour-associated macrophages and myeloid-derived suppressor cells via NKT T cell receptor recognition, actively remodelling the RCC TME [116]. Notably, AlloCAR70-NKT cells eliminate CD70^+^ host alloreactive T cells while sparing CD70^−^ populations, promoting therapeutic persistence and mitigating graft-versus-host responses. In orthotopic and metastatic xenograft models, AlloCAR70-NKT cells demonstrated superior tumour homing, enhanced effector function, reduced exhaustion, and a favourable safety profile, with minimal cytokine release syndrome and organ toxicity compared to conventional CAR70-T cells [117]. A detailed clinically guided protocol has been established for generating these cells from cord blood CD34^+^ haematopoietic stem and progenitor cells, encompassing five sequential ex vivo differentiation stages and achieving yields of up to 1 × 10^12^ cells from a single cord blood unit, sufficient to treat thousands of patients [118]. These findings support AlloCAR70-NKT cells as a next-generation, off-the-shelf immunotherapy with dual tumour- and TME-targeting functionality and the added capacity to overcome allorejection, offering a compelling strategy for treating metastatic RCC.

**Figure 2:**
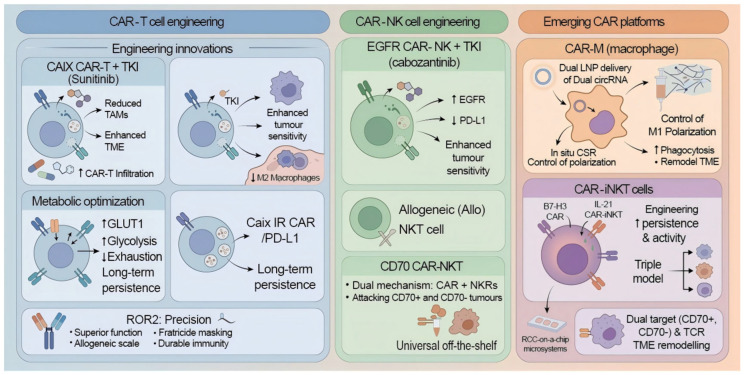
Engineering innovations and emerging platforms for CAR-based cell therapies in RCC. This figure summarises key engineering strategies (affinity tuning, cytokine armouring, metabolic enhancement, and combination approaches) and emerging platforms (CAR-M, CAR-iNKT, γδ CAR-T) discussed in Section 3.1, Section 3.2 and Section 3.3. Abbreviations: CAR, chimeric antigen receptor; CAR-M, CAR-macrophage; CAIX, carbonic anhydrase IX; circRNA, circular RNA; CRISPR, clustered regularly interspaced short palindromic repeats; EGFR, epidermal growth factor receptor; GLUT1, glucose transporter type 1; iNKT, invariant natural killer T cell; LNP, lipid nanoparticle; NKR, natural killer receptor; PD-L1, programmed death ligand 1; RCC, renal cell carcinoma; ROR2, receptor tyrosine kinase-like orphan receptor 2; TKI, tyrosine kinase inhibitor; TME, tumour microenvironment.

Collectively, these emerging platforms, CAR-M and CAR-iNKT cells, expand the repertoire of CAR-based immunotherapies for RCC beyond conventional T and NK cells. CAR-M offers a unique mechanism of action centred on phagocytosis and TME remodelling, with synthetic cytokine receptor engineering enabling precise control of the polarisation state [119]. CAR-iNKT cells combine the advantages of CAR-dependent targeting with innate-like recognition via natural killer receptors, enabling activity against antigen-low or antigen-negative tumours while actively depleting immunosuppressive myeloid populations. Both platforms have demonstrated robust preclinical efficacy in orthotopic and metastatic RCC models, with favourable safety profiles supporting continued development toward clinical translation. To date, CAR-NK/CAR-M/CAR-iNKT platforms for RCC remain at the preclinical stage of development, with no registered clinical trials for CAR-M or CAR-iNKT and only one phase I/II trial for CAR-NK. The emerging platforms and next-generation engineering innovations discussed in this section are overviewed in Figure 2. A comprehensive comparison of CAR-T, CAR-NK, and CAR-M/CAR-iNKT platforms is presented in Table 1.

### 3.4. Mechanisms of Resistance to CAR Cell Therapy in RCC

Despite promising preclinical activity, durable responses to CAR cell therapy in RCC remain rare. Resistance can be classified as intrinsic (pre-existing) or acquired (developed after treatment).

Intrinsic resistance. Pre-existing factors include (1) low or heterogeneous target antigen expression—CD70, CAIX, or B7-H3 levels vary across lesions and subclones, limiting CAR recognition [122]; (2) physical barriers—CAF-rich stroma and abnormal vasculature impede CAR cell infiltration [123]; (3) immunosuppressive TME—TAMs, MDSCs, and Tregs suppress CAR cell function from the outset [122]; (4) metabolic stress—hypoxia, acidosis, and nutrient depletion impair CAR cell fitness [61]; and (5) host immunogenicity—murine CAR components can elicit anti-CAR immune responses, limiting persistence [124].

Acquired resistance. Mechanisms emerging after CAR exposure include (1) antigen escape—selection of CD70-low or CAIX-negative clones under CAR pressure (discussed in Section 3.1) [125]; (2) CAR T cell exhaustion—chronic activation upregulates PD-1, TIM-3, and LAG-3, leading to functional decline [126]; (3) upregulation of alternative checkpoints—TIGIT, VISTA, or other inhibitory molecules may compensate for PD-1 blockade [127]; (4) expansion of suppressive populations—Tregs and MDSCs may increase following CAR-T infusion [128]; and (5) loss of CAR persistence—limited in vivo expansion and contraction within weeks, as observed in the COBALT-RCC trial [126].

Understanding these resistance mechanisms is essential for designing next-generation strategies. Approaches to overcome them—including combinatorial targeting, switch receptors, metabolic engineering, and allogeneic platforms—are discussed in Section 5.

## 4. CAR-Based Therapies for Renal Cell Carcinoma: Evidence from Clinical Trials

The first-in-human experience, reported by Lamers and colleagues at Erasmus MC, targeted CAIX in twelve patients with metastatic clear cell RCC using a first-generation CAR derived from the G250 monoclonal antibody [129]. While circulating CAR-T cells were detectable and retained antigen-specific function ex vivo, no objective clinical responses were observed. More critically, dose-limiting on-target off-tumour toxicity manifested as grade 2–4 liver enzyme elevations in four of eight patients treated without protective measures. Histological examination revealed CAIX expression on the biliary duct epithelium accompanied by T cell infiltration, directly confirming the mechanistic basis of this toxicity. Subsequent cohort administration of the parental G250 monoclonal antibody prior to CAR-T infusion successfully blocked hepatic CAIX sites and mitigated liver injury, yet it did not improve the therapeutic efficacy [130]. This trial also uncovered a further barrier: the murine-derived single-chain variable fragment of the CAR proved highly immunogenic, eliciting both humoral and cellular anti-CAR immune responses that limited T cell persistence to weeks rather than months [131]. These findings established that target selection, affinity tuning, and immunogenicity reduction are prerequisites for clinical success.

Building on these lessons, the CD70 antigen has emerged as a more tractable target, given its high and stable expression in over 80% of ccRCCs and its more restricted normal tissue distribution [132]. The phase I COBALT-RCC trial evaluated CTX130, an allogeneic, CRISPR-Cas9-edited CAR-T cell product targeting CD70, in sixteen heavily pretreated patients with relapsed or refractory ccRCC. Notably, the product incorporated three strategic edits: the insertion of the anti-CD70 CAR cassette into the T cell receptor alpha constant (TRAC) locus to eliminate TCR expression and mitigate the graft-versus-host disease risk; the disruption of β2-microglobulin to reduce MHC-mediated rejection; and, most innovatively, CD70 knockout to prevent CAR-T cell fratricide. The safety profile was favourable, with no dose-limiting toxicities and only grade 1–2 cytokine release syndrome occurring in half of the patients, without any instances of immune effector cell-associated neurotoxicity syndrome or graft-versus-host disease [133]. Disease control was achieved in 81.3% of patients, and, notably, one patient continues in a durable complete response at three years after infusion, representing the first such sustained remission achieved with a CAR-T cell product in any solid tumour. Pharmacokinetic analyses revealed rapid initial expansion followed by contraction, with CAR-T cells typically becoming undetectable by day 28, highlighting persistence as an ongoing limitation. The next-generation product CTX131 (NCT05795595), with additional Regnase-1 and TGFβR2 knockouts, is now advancing toward clinical evaluation [12].

An orthogonal approach has been explored by Chen and colleagues, who engineered autologous tumour-infiltrating lymphocytes to co-express a PD-1-CD28 switch receptor and a CD19 CAR, termed S-TILs, aiming to simultaneously reverse checkpoint-mediated inhibition and provide CAR-driven expansion upon encountering CD19-positive B cells. In a small phase I trial that included one patient with metastatic kidney cancer, this strategy proved safe, without grade 3–5 adverse events, and the renal cancer patient achieved stable disease at three months following rapid disease progression prior to treatment [134]. While the therapeutic efficacy of CAR-T cells in RCC remains inferior to that achieved in haematological malignancies, the durable complete response observed with CD70-directed therapy provides formal proof of concept that this approach can produce a meaningful clinical benefit [13]. The convergence of optimised target selection, multi-edited allogeneic products, and rational combination strategies, including tyrosine kinase inhibitors and checkpoint blockade, now provides a clear roadmap for the next generation of clinical trials in this disease [135]. The clinical evaluation of CAR-based therapies in renal cell carcinoma has entered an era of diversification. Following the foundational but clinically limited first-in-human experience with CAIX-directed CAR-T cells, the field has strategically expanded to address the core barriers of target suitability, on-target off-tumour toxicity, cellular persistence, and the immunosuppressive tumour microenvironment [136].

The most advanced clinical data currently available pertain to CD70-directed therapies. The COBALT-RCC trial (NCT04438083) evaluated CTX130, an allogeneic CRISPR-Cas9-edited CAR-T cell product, in sixteen heavily pretreated patients with relapsed or refractory ccRCC. This study demonstrated a manageable safety profile with no dose-limiting toxicities, achieved disease control in 81.3% of patients, and produced a durable complete response now exceeding three years—the first such sustained remission reported for any CAR-T cell product in a solid tumour. Building on this proof of concept, a next-generation product, CTX131 (NCT05795595), has entered clinical evaluation, with additional Regnase-1 and TGFβR2 knockouts designed to enhance functional persistence and counteract immunosuppressive signals within the RCC microenvironment.

Multiple other CD70-directed autologous CAR-T trials are actively recruiting. A phase I study (NCT05420519) is evaluating CD70-targeted CAR-T cells in patients with advanced or metastatic CD70-positive RCC following standard lymphodepleting chemotherapy with fludarabine and cyclophosphamide. A parallel study (NCT05420545) is investigating a similar CD70-directed autologous CAR-T product in CD70-positive advanced solid tumours, including RCC. Additional CD70-targeted studies include NCT05518253, NCT06010875, NCT05468190, NCT06586658, NCT07297667, NCT04897321, NCT04696731, NCT05239143, NCT06682793, NCT07500805, NCT06245915, NCT06383507, and NCT05672459, reflecting the substantial clinical interest in this target.

An innovative approach to CD70 targeting employs the γδ T cell platform. ADI-270 (NCT06480565) is a first-in-human phase I/II trial of an allogeneic γδ CAR-T cell product targeting CD70, engineered with a dominant-negative TGFβ receptor II to confer resistance to the immunosuppressive tumour microenvironment. The product utilises the natural CD27 receptor as the binding moiety, potentially offering distinct binding kinetics and reduced immunogenicity compared to scFv-based constructs. A separate Vδ1 CAR-T cell study (NCT07113977) further expands this platform.

The CAR-NKT platform has also entered clinical evaluation. A phase I study of CD70-targeted CAR-NKT cells (CGC738, NCT06870279) is enrolling patients with advanced ccRCC following lymphodepleting chemotherapy with fludarabine and cyclophosphamide.

The natural killer cell platform offers a distinct safety and logistical profile. A phase I/II study of CD70-directed cord blood-derived CAR-NK cells engineered to express IL-15 (NCT05703854) is recruiting patients with advanced RCC, with the IL-15 transgene specifically designed to address the historically poor persistence of adoptively transferred NK cells. Additional CAR-NK trials for RCC (NCT07072234, NCT07410676) are now active.

The historically challenging CAIX target has been revisited with improved safety measures. A phase I trial (NCT04969354) has administered CAIX-directed CAR-T cells following hepatic artery infusion of the parental G250 monoclonal antibody to block antigen expression on the biliary epithelium, directly addressing the on-target off-tumour toxicity that halted earlier studies. Another CAIX-directed study (NCT03638206) is also ongoing.

Other targets under clinical investigation include ROR2 and AXL through an adaptive phase I/II design (NCT03393936), assigning patients to CCT301-59 (ROR2-positive) or CCT301-38 (AXL-positive, ROR2-negative) based on the tumour biopsy profile. Additional studies targeting PSMA (NCT07297160) and VEGFR2 (NCT01218867), as well as multi-target strategies (NCT07181720), further enrich the pipeline, along with NCT02830724 (CD70-binding CAR transduced peripheral blood lymphocytes).

Collectively, this extensive portfolio of registered clinical trials marks a definitive transition from exploratory first-in-human studies to a new era of rational, mechanism-based cellular therapy development for RCC. The convergence on validated targets such as CD70; the diversification across distinct cellular platforms, including autologous and allogeneic CAR-T, γδ T cells, CAR-NKT cells, and CAR-NK cells; and the incorporation of sophisticated engineering solutions such as cytokine armouring, switch receptors, conditional culture methods, and TGFβ resistance provide a clear roadmap for the field. The results of these studies, anticipated over the coming years, will determine which of these innovative platforms can translate compelling preclinical activity into a meaningful, durable clinical benefit for patients with advanced RCC. Table 2 provides a detailed summary of these ongoing and completed trials, including NCT numbers, therapy types, targets, RCC-relevant indications, phases, and recruitment statuses.

## 5. Future Directions for CAR-Based Therapies in Renal Cell Carcinoma

**Figure 3:**
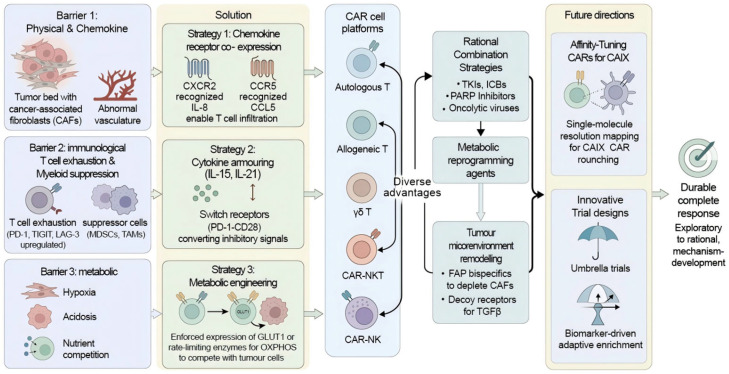
Overcoming barriers to CAR cell therapy in solid tumours: strategies and future directions. This figure integrates the three major TME barriers (physical, immunological, and metabolic) with corresponding engineering strategies (chemokine receptor co-expression, switch receptors, cytokine armouring, metabolic reprogramming, and combination therapies) proposed to overcome them. Abbreviations: CAF, cancer-associated fibroblast; CAR, chimeric antigen receptor; CCR5, C-C chemokine receptor type 5; CXCR2, C-X-C chemokine receptor type 2; FAP, fibroblast activation protein; GLUT1, glucose transporter type 1; ICB, immune checkpoint blockade; IL, interleukin; LAG-3, lymphocyte activation gene 3; MDSC, myeloid-derived suppressor cell; OXPHOS, oxidative phosphorylation; PARP, poly (ADP-ribose) polymerase; PD-1, programmed cell death protein 1; TAM, tumour-associated macrophage; TGFβ, transforming growth factor beta; TIGIT, T cell immunoreceptor with Ig and ITIM domains; TKI, tyrosine kinase inhibitor.

Collectively, the registered trials mark a transition from exploratory studies to mechanism-based cellular therapy development for RCC. However, the durable complete response observed with CTX130 remains exceptional. The path forward requires addressing three interconnected barriers: physical (CAFs, abnormal vasculature), immunological (exhaustion, myeloid suppression), and metabolic (hypoxia, acidosis, nutrient competition) [137,138,139]. The interconnections between these three barriers and the corresponding engineering strategies designed to overcome them are illustrated schematically in Figure 3.

Toxicity considerations in RCC. CRS and ICANS, while well-characterised in haematologic malignancies, appear less frequent and severe in RCC CAR-T trials. In the COBALT-RCC trial, only grade 1–2 CRS was observed, with no ICANS, suggesting that solid tumour CAR-T may have a more favourable acute toxicity profile [140]. However, on-target/off-tumour toxicity remains a critical concern, as demonstrated by the CAIX G250 CAR trial, where CAIX expression on the biliary epithelium caused dose-limiting cholangitis. Unlike haematologic targets (e.g., CD19), which are largely restricted to B cells, solid tumour antigens often have low-level expression on vital tissues, necessitating rigorous preclinical safety assessment and affinity tuning [72].

The dominance of CD70 as the clinical target of choice is now undeniable. With over thirty registered trials targeting this antigen across autologous CAR-T, allogeneic CAR-T, γδ CAR-T, CAR-NKT, and CAR-NK platforms, CD70 has become the CD19 of RCC immunotherapy [141].

Several engineering strategies currently in preclinical development warrant accelerated clinical translation. First, cytokine armouring, particularly IL-15 and IL-21 transgenes, has shown exceptional promise in promoting T and NK cell persistence while preserving a less differentiated, stem-like memory phenotype [142,143]. The ongoing clinical evaluation of IL-15-armoured CAR-NK cells (NCT05703854) will provide an early readout of this strategy in RCC. Second, chemokine receptor co-expression to match tumour-derived chemokines, such as CXCR2 to recognise IL-8 or CCR5 to recognise CCL5, could overcome the physical exclusion of CAR-T cells from tumour nests [144]. Third, switch receptors that convert inhibitory signals (PD-1, TIGIT, LAG-3) into activating signals (CD28, 4-1BB) offer a dual benefit of checkpoint blockade and signal reversal, with PD-1-CD28 constructs already entering clinical testing in the S-TILs platform [145]. Fourth, metabolic engineering—enforced expression of GLUT1 or rate-limiting enzymes in oxidative phosphorylation—may enable CAR-T cells to outcompete tumour cells for glucose and glutamine, directly countering the Warburg effect that characterises ccRCC [146]. Is metabolic enhancement sufficient? While GLUT1 overexpression or PIM3 inhibition can improve CAR-T metabolic fitness [147], it remains uncertain whether these strategies alone can overcome the profoundly hypoxic and nutrient-depleted ccRCC microenvironment. ccRCC exhibits extreme metabolic reprogramming (Warburg effect, glutamine addiction) that enables tumour cells to outcompete CAR-T cells [148]. Hypoxia directly induces CAR-T exhaustion and apoptosis through HIF-1α pathways—effects that may not be fully reversible by metabolic enhancement alone. Therefore, metabolic engineering should be a component of a multi-pronged strategy—paired with hypoxia-sensing CARs, chemokine receptors, or HIF-2α inhibitors—rather than a standalone solution.

The tumour microenvironment in RCC is not a passive bystander but an active driver of resistance. The single-cell and spatial transcriptomic analyses described earlier in this review have revealed that CAF subsets, TAM states, and exhaustion trajectories exist as functional continua that evolve with therapy. Future CAR-based therapies must therefore be dynamic rather than static. One approach is to engineer CAR-T cells to secrete tumour microenvironment-remodelling factors, such as anti-PD-L1 antibodies (as demonstrated in the G36-PDL1 construct), FAP-targeting bispecifics to deplete CAFs, or decoy receptors for TGFβ to neutralise this dominant immunosuppressive cytokine. Another approach is to design CARs with tuneable activity, using small-molecule on-switches or hypoxia-sensing promoters to restrict activation to the tumour bed, thereby mitigating on-target off-tumour toxicity and potentially enabling the safe targeting of antigens with broader normal tissue expression.

The diversification across cellular platforms, autologous CAR-T, allogeneic CAR-T, γδ CAR-T, CAR-NKT, and CAR-NK, is a strength of the current pipeline, but head-to-head comparisons in preclinical models and early-phase trials are urgently needed. Each platform offers distinct advantages: allogeneic products enable off-the-shelf availability and cost reduction; γδ T cells and NK cells offer innate tumour recognition and low graft-versus-host disease risks; and CAR-NKT cells combine CAR-dependent and NKR-dependent killing, potentially overcoming antigen escape. However, each also has unique limitations. The optimal platform may ultimately differ by patient subset, disease stage, and prior treatment history, suggesting that a portfolio of products rather than a single winner will characterise the mature field.

Manufacturing, economic, and regulatory barriers. Beyond biological and engineering challenges, practical barriers impede widespread implementation. Logistically, autologous CAR-T requires centralised GMP facilities and 2–4 weeks of manufacturing, which may be unsuitable for rapidly progressing disease; allogeneic products face host rejection and limited persistence. Economically, CAR-T costs exceed USD 400,000 per patient, requiring cost-effectiveness analyses given the existing lower-cost options (TKIs, ICBs) for RCC. Regulatory barriers include establishing lot release criteria for gene-edited products, defining acceptable persistence thresholds, and standardising potency assays. Addressing these barriers will require collaboration among industry, regulators, and payers.

Combination strategies will be essential. The preclinical synergy between CAR-T cells and tyrosine kinase inhibitors such as sunitinib and cabozantinib, discussed earlier in this review, provides strong rationale for clinical testing [149]. TKIs offer dual benefits: they remodel the tumour microenvironment by reducing MDSCs and TAMs, and they upregulate target antigen expression on tumour cells [150]. Similarly, the combination of CAR-T cells with immune checkpoint blockade is mechanistically compelling, as CAR-T cell activation upregulates PD-1 and other exhaustion markers, rendering them susceptible to PD-1/PD-L1 inhibition. Beyond TKIs and ICBs, emerging combinations include PARP inhibitors (which activate the cGAS-STING pathway and promote chemokine-driven CAR-T infiltration), oncolytic viruses, and metabolic modulators [151].

The lessons from CAIX-directed therapy must inform future target selection. The failure of the G250 CAR was not due to inadequate target density on tumours—CAIX is abundantly expressed on ccRCC—but rather due to low-level expression on the normal bile duct epithelium [152]. This highlights a general principle: for solid tumours, the target density on tumour cells must be balanced against the activation threshold. High-affinity CARs may be too sensitive, recognising low-density antigens on normal tissues and causing on-target off-tumour toxicity [153]. Affinity tuning, as demonstrated for the G9 CAIX CAR, offers a path forward, but this approach requires detailed knowledge of the antigen density on both tumour and normal tissues at a single-molecule resolution. The RCC community should therefore prioritise quantitative antigen mapping across all potential targets [154,155].

Finally, the design of clinical trials for next-generation CAR-based therapies in RCC must evolve. The standard 3+3 dose escalation design is poorly suited to cellular therapies, where toxicity is not dose-dependent in a linear fashion and efficacy may plateau at sub-toxic doses [156]. Alternative designs, including Bayesian optimal interval designs and umbrella trials that assign patients to different CAR products based on tumour biomarkers, should be adopted. The adaptive design of the ROR2/AXL trial (NCT03393936) provides a model for biomarker-driven enrichment. Furthermore, the field must move beyond RECIST response rates as the primary endpoint. CAR-T therapies can produce prolonged disease stabilisation without explicit tumour shrinkage, as seen in the S-TILs trial. Progression-free survival, the duration of response, and patient-reported outcomes should be prioritised alongside objective response rates [157]. Recommendations for trial design. Endpoints: Include PFS, DOR, and CR rate alongside ORR; incorporate PROs. Biomarkers: Mandatory pretreatment biopsies for target expression (CD70, CAIX, B7-H3) and heterogeneity; assess PD-L1, TME features, and mutational status (PBRM1, BAP1); monitor CAR-T persistence and ctDNA; post-progression biopsies. Patient populations: Prioritise relapsed/refractory after ≥1 line of ICI/TKI; high target expression (>70%) and low tumour burden preferred. Combinations: Prioritise CAR-T + TKI (cabozantinib), CAR-T + PD-1/PD-L1 inhibitor, CAR-T + PARP inhibitor, and CAR-T + HIF-2α inhibitor. Explore sequential dosing to reduce toxicity.

In conclusion, the future of CAR-based therapy for RCC holds promise but faces substantial challenges. The convergence of optimised target selection, cellular engineering, combination strategies, and biomarker-driven trial design provides a framework for continued development. The next five years will determine whether the durable complete response achieved with CTX130 represents a rare outlier or the beginning of a more consistent clinical benefit. Significant engineering and biological barriers remain, and achieving a cure remains an aspirational goal rather than an imminent reality based on the current evidence.

## 6. Discussion

In summary, CAR-based therapies have emerged as a legitimate therapeutic modality for renal cell carcinoma, moving from a theoretical concept to clinical proof of concept. The durable complete response achieved with CD70-directed CTX130 in the COBALT-RCC trial—now exceeding three years—provides formal validation that cellular immunotherapy can produce a meaningful, long-lasting benefit in a solid tumour [12]. This notable observation—the first of its kind for any CAR-T cell product in a solid malignancy—demonstrates that the barriers to CAR therapy in RCC are surmountable.

However, as this body of evidence makes clear, such responses remain exceptional rather than routine. Among sixteen heavily pretreated patients in the COBALT-RCC trial, only one achieved a complete response, and CAR-T cells typically became undetectable by day 28 after infusion [12]. The first-in-human CAIX-directed trial produced no objective responses and was limited by on-target off-tumour toxicity and CAR immunogenicity. These outcomes underscore that major barriers persist. The RCC tumour microenvironment imposes three interconnected obstacles that collectively limit therapeutic efficacy: the physical barrier of cancer-associated fibroblasts and abnormal vasculature, which excludes T cells from tumour nests; the immunological barrier of T cell exhaustion and myeloid suppression, which renders infiltrating T cells dysfunctional; and the metabolic barrier of hypoxia, acidosis, and nutrient competition, which directly impairs T cell metabolism and function. No single engineering strategy is likely to overcome all three simultaneously [158,159].

The lessons from early failures have fundamentally reshaped the field. CAIX taught that target selection must be balanced against the activation threshold, that affinity tuning is not optional but essential, and that immunogenicity reduction is a prerequisite for clinical success [160]. CD70 has since become the dominant clinical target, with over thirty registered trials across autologous CAR-T, allogeneic CAR-T, γδ CAR-T, CAR-NKT, and CAR-NK platforms. Yet the heterogeneity of RCC—across patients, across metastatic sites within the same patient, and across subclones within the same tumour—demands a portfolio of approaches rather than convergence on a single target or platform [161].

The path forward lies in next-generation engineering strategies that simultaneously address the physical, immunological, and metabolic barriers of the TME. Cytokine armouring with IL-15 or IL-21 enhances persistence while preserving a stem-like memory phenotype [162]. Chemokine receptor co-expression overcomes physical exclusion by guiding CAR-T cells along chemokine gradients. Switch receptors that convert PD-1, TIGIT, or LAG-3 signals into CD28 or 4-1BB activation reverse exhaustion while providing intrinsic checkpoint blockade. Metabolic reprogramming through enforced GLUT1 expression enables T cells to outcompete tumour cells for glucose and glutamine, directly countering the Warburg effect that characterises ccRCC [163]. The diversification across cellular platforms—allogeneic CAR-T, γδ T cells, NK cells, NKT cells, and macrophages—offers multiple paths to success, each with distinct advantages and limitations. Head-to-head comparisons in preclinical models and early-phase trials are urgently needed.

Combination strategies will be essential. The preclinical synergy between CAR-T cells and tyrosine kinase inhibitors, discussed earlier in this review, provides strong rationale for clinical testing. TKIs remodel the TME by reducing MDSCs and TAMs while upregulating target antigen expression. Similarly, combining CAR-T cells with immune checkpoint blockade is mechanistically compelling, as CAR-T cell activation upregulates PD-1 and other exhaustion markers. Beyond TKIs and ICBs, emerging combinations include PARP inhibitors, oncolytic viruses, and metabolic modulators [164].

Finally, the design of clinical trials must evolve. The standard 3+3 dose escalation design is poorly suited to cellular therapies. Alternative designs, including Bayesian optimal interval designs and biomarker-driven umbrella trials, should be adopted. The adaptive design of the ROR2/AXL trial (NCT03393936) provides a model. Furthermore, the field must move beyond RECIST response rates as the primary endpoint. Prolonged disease stabilisation without clear tumour shrinkage, as seen in the S-TILs trial, can represent a meaningful clinical benefit [165].

Limitations of current evidence. It is important to acknowledge that the evidence supporting these projections remains limited. To date, only one durable complete response has been reported across all CAR-based trials in ccRCC (CTX130 in the COBALT-RCC trial), and CAR-T cell persistence beyond 28 days has not been consistently achieved. Most registered trials are in early phases, and no CAR-based product has received regulatory approval for RCC. Therefore, while the concept of a cure is a legitimate long-term goal, it remains speculative at this stage, and near-term expectations should focus on achieving meaningful disease control and improved quality of life.

## 7. Conclusions

In summary, CAR-based therapies have transitioned from a theoretical concept to clinical proof of concept in renal cell carcinoma. The durable complete response exceeding three years achieved with CD70-directed CTX130 in the COBALT-RCC trial provides formal validation that cellular immunotherapy can produce a meaningful, long-lasting benefit in a solid tumour. However, such responses remain exceptional rather than routine, underscoring that major barriers persist within the ccRCC tumour microenvironment—namely the interconnected physical, immunological, and metabolic obstacles that collectively limit the therapeutic efficacy.

CD70 has emerged as the dominant clinical target, with over thirty registered trials across autologous CAR-T, allogeneic CAR-T, γδ CAR-T, CAR-NKT, and CAR-NK platforms. Nevertheless, the heterogeneity of ccRCC demands a portfolio of approaches rather than convergence on a single platform. Next-generation engineering strategies that simultaneously address TME barriers, including cytokine armouring, chemokine receptor co-expression, switch receptors, and metabolic reprogramming, are essential for improving patient outcomes.

The path forward lies in rational combination strategies, biomarker-driven trial design, and the continued integration of single-cell and spatial technologies. However, significant engineering and biological barriers remain. With continued advances, the field may eventually improve outcomes for patients with ccRCC, but achieving a cure remains an aspirational goal based on the current evidence.

## Figures and Tables

**Table 1:** Comparison of CAR-engineered cell therapies in RCC.

Dimension	CAR-T Cells	CAR-NK Cells	CAR-M/CAR-iNKT Cells
Main Targets	B7-H3 [69], CAIX [80], CD70 [12], ROR2 [89], c-Met [90]	EGFR [101], CAIX, CD70 [98]	B7H3 [114], CD70 [115]
Source and Accessibility	Mainly autologous, off-the-shelf limited	Allogeneic sources (cord blood, NK-92, iPSCs), off-the-shelf feasible	Allogeneic sources (monocytes/macrophages, cord blood HSPCs), off-the-shelf feasible (thousands of doses per unit)
Advantages and Limitations	Advantages: High specificity, durable persistence, solid clinical validation	Advantages: Off-the-shelf potential, low CRS/GVHD, innate NKR activity	Advantages: Overcomes antigen escape, actively remodels TME
	Limitations: OTOT toxicity, TME suppression, long manufacturing time [120]	Limitations: Poor in vivo persistence, weak expansion capacity [95]	Limitations: CAR-M prone to M2 polarisation; CAR-iNKT complex manufacturing [111]
Key Functional Differences	Antigen escape response: Weak [65] (single CAR dependent)	Antigen escape response: Moderate [98] (CAR + NKR dual recognition)	Antigen escape response: Strong (CAR + NKR + TCR multi-recognition)
(Escape + TME)	TME modulation: Weak (easily suppressed)	TME modulation: Moderate (can combine with drugs)	TME modulation: Strong (actively depletes M2/TAM/MDSC)
Core Engineering Strategies	Affinity tuning (G9 for CAIX)	TKI combination (cabozantinib) for sensitisation	CAR-M: CSR synthetic receptor + LNP delivery + hydrogel [121]
	Anti-PD-L1 secretion (G36-PDL1)	Bortezomib combination	CAR-iNKT: IL-21 co-expression + multi-stage differentiation [114]
	Metabolic enhancement [65] (GLUT1)	Irradiation for safety [98]	
Clinical Progress	Leading (early clinical trials, e.g., ADI-270, NCT06480565)	Predominantly preclinical	Preclinical (with clear translational protocols, e.g., AlloCAR70-NKT)

Note: CAR, chimeric antigen receptor; CRS, cytokine release syndrome; CSR, chimeric signalling receptor; EGFR, epidermal growth factor receptor; GVHD, graft-versus-host disease; GLUT1, glucose transporter 1; HSPC, haematopoietic stem and progenitor cell; IL-21, interleukin-21; iPSC, induced pluripotent stem cell; LNP, lipid nanoparticle; MDSC, myeloid-derived suppressor cell; NKR, natural killer receptor; OTOT, on-target off-tumour; PD-L1, programmed death ligand 1; RCC, renal cell carcinoma; TAM, tumour-associated macrophage; TCR, T cell receptor; TKI, tyrosine kinase inhibitor; TME, tumour microenvironment.

**Table 2:** Summary of ongoing and completed clinical trials of CAR-based therapies for renal cell carcinoma.

NCT Number	Therapy Type	Target	Indication (RCC Focus)	Phase	Status
NCT07181720	CAR-T	CD70	RCC, cervical, ovarian, lung	I	Recruiting
NCT04969354	CAR-T	CAIX	RCC	I	Recruiting
NCT03638206	CAR-T	Multi-target	Multiple tumours (incl. RCC)	I/II	Unknown
NCT03393936	CAR-T	ROR2	RCC	I/II	Terminated
NCT07297160	CAR-T	CD70 (CALvSSEY)	CD70+ lymphoma, myeloma, solid tumours	I	Not yet recruiting
NCT05420519	CAR-T	CD70	RCC only (IHC 3+)	I	Unknown
NCT02830724	CAR-T	CD70 (CD27-based)	CD70+ cancers (RCC, pancreatic, breast, melanoma, ovarian)	I/II	Recruiting
NCT06010875	CAR-T	CD70	RCC, ovarian, cervical	I	Recruiting
NCT05103631	CAR-T	GPC3 + IL-15	GPC3+ solid tumours (HCC, Wilms, rhabdomyosarcoma)	I	Active, not recruiting
NCT05518253	CAR-T	CD70	RCC, ovarian, cervical	I	Recruiting
NCT05420545	CAR-T	CD70	RCC, ovarian, cervical	I	Unknown
NCT05468190	CAR-T	CD70	RCC, ovarian, cervical	I	Unknown
NCT06586658	CAR-T	CD70	Locally advanced/inoperable RCC	I	Recruiting
NCT07297667	CAR-T	GPNMB (GCAR1)	RCC, TNBC, HNSCC	I	Recruiting
NCT04897321	CAR-T	B7-H3 (3CAR)	Paediatric solid tumours (osteosarcoma, neuroblastoma, Wilms, etc.)	I	Recruiting
NCT04696731	Allogeneic CAR-T	CD70 (ALLO-316; TALEN)	R/R ccRCC (post ICI/VEGF)	I	Active, not recruiting
NCT05239143	Allogeneic CAR-T	MUC1 (P-MUC1C-ALLO1)	Breast, ovarian, pancreatic, CRC, NSCLC, RCC, HNSCC, etc.	I	Active, not recruiting
NCT04438083	Allogeneic CAR-T	CD70 (CTX130;CRISPR)	R/R ccRCC (post CPI/TKI)	I	Terminated
NCT06682793	Allogeneic CAR-T	Logic-gated (EGFR activator/HLA-A*02 blocker)	EGFR+ solid tumours with HLA LOH (CRC, NSCLC, HNSCC, TNBC, RCC)	I/II	Recruiting
NCT07500805	CAR-T	CD70 (Anti-CD70 UCAR-T)	CD70+ advanced ccRCC	I	Not yet recruiting
NCT01218867	CAR-T	VEGFR2	RCC, metastatic melanoma	I/II	Terminated
NCT06245915	CAR-T	CAIX + PSMA (logic gate, AND gate)	R/R ccRCC (post CPI/VEGF)	I/II	Active, not recruiting
NCT05795595	Allogeneic CAR-T	CD70 (CTX131, CRISPR)	ccRCC, cervical, pancreatic, oesophageal, mesothelioma	I/II	Completed
NCT06383507	CAR-T	CD70 (CHT101)	RCC, ovarian, cervical, HNSCC, nasopharyngeal	I	Not recruiting
NCT05672459	CAR-T (3rd gen)	HLA-G	HLA-G+ solid tumours (RCC, ovarian)	I/IIa	Active, not recruiting
NCT06480565	Allogeneic CAR Vδ1 γδ T cell	CD70 (ADI-270, dnTGFβRII)	R/R ccRCC (post ICI/VEGF)	I/II	Active, not recruiting
NCT07113977	CAR-T	CD70	Advanced RCC	I	Not yet recruiting
NCT06870279	CAR-NKT	CD70 (CGC738)	Advanced ccRCC	I	Not yet recruiting
NCT07072234	CAR-NK	CD70 (TGFβR2 KO)	Treatment-refractory ccRCC	I	Recruiting
NCT05703854	CAR-NK	CD70 (CB-derived, IL15-transduced)	Advanced RCC, mesothelioma, osteosarcoma	I/II	Recruiting
NCT07410676	Allogeneic NK cells (CAR-NK implied)	CD70 (EBNK-001, ±pembrolizumab)	Multiple solid tumours (RCC, sarcoma, GBM, CRC, ovarian, pancreatic, lung, etc.)	I	Recruiting

Abbreviations: CAR, chimeric antigen receptor; ccRCC, clear cell renal cell carcinoma; CRC, colorectal cancer; GBM, glioblastoma; HNSCC, head and neck squamous cell carcinoma; ICI, immune checkpoint inhibitor; LOH, loss of heterozygosity; NSCLC, non-small cell lung cancer; RCC, renal cell carcinoma; R/R, relapsed/refractory; TNBC, triple-negative breast cancer; VEGF, vascular endothelial growth factor; TKI, tyrosine kinase inhibitor; UCAR-T, universal chimeric antigen receptor T cell. Note: Data retrieved from ClinicalTrials.gov (as of April 2026). Status definitions: “Recruiting” = actively enrolling; “Active, not recruiting” = study in progress but not accepting new patients; “Not yet recruiting” = not yet open for enrolment; “Unknown status” = status not updated in past 2 years; “Terminated” = stopped early; “Completed” = study finished.

## Data Availability

No new data were created or analyzed in this study.

## Abbreviations

The following abbreviations are used in this manuscript:

ADCC	antibody-dependent cellular cytotoxicity
AlloCAR70-NKT	CD70-directed allogeneic CAR-NKT cells
apCAFs	antigen-presenting cancer-associated fibroblasts
CAFs	cancer-associated fibroblasts
CAIX	carbonic anhydrase IX
CAR	chimeric antigen receptor
CAR-NKT	CAR-engineered natural killer T cells
circRNAs	circular RNAs
COX-2	cyclooxygenase-2
CSR	chimeric interleukin-2 signalling receptor
DCs	dendritic cells
dnTGFβRII	dominant-negative transforming growth factor beta receptor II
dSTORM	direct stochastic optical reconstruction microscopy
EGFR	epidermal growth factor receptor
EMT	epithelial–mesenchymal transition
GVHD	graft-versus-host disease
HSPCs	haematopoietic stem and progenitor cells
iCAFs	inflammatory cancer-associated fibroblasts
ICB	immune checkpoint blockade
IDO	indoleamine 2,3-dioxygenase
IL-2	interleukin-2
iNKT	invariant natural killer T cells
iPSCs	induced pluripotent stem cells
IR	immune-restoring
LDH	lactate dehydrogenase
LNPs	lipid nanoparticles
LOX	lysyl oxidase
MDSCs	myeloid-derived suppressor cells
MMPs	matrix metalloproteinases
myCAFs	myofibroblastic cancer-associated fibroblasts
NK	natural killer
NKR	natural killer receptor
NKRs	natural killer receptors
NKT	natural killer T cells
OTOT	on-target off-tumour
PD-L1	programmed death ligand 1
PDOTS	patient-derived organotypic tumour spheroid
PDX	patient-derived xenograft
PRCC	papillary renal cell carcinoma
Tfh	T follicular helper cell
TKIs	tyrosine kinase inhibitors
TLS	tertiary lymphoid structure
TME	tumour microenvironment
TRAC	T cell receptor alpha constant
Treg	regulatory T cell
VEGF	vascular endothelial growth factor
VHL	von Hippel–Lindau
α-GC	α-galactosylceramide
